# Epidemiology, Pathogenesis, and Diagnosis of Cardiac Sarcoidosis

**DOI:** 10.14797/mdcvj.1057

**Published:** 2022-03-14

**Authors:** Sheetal V. Mathai, Snehal Patel, Ulrich P. Jorde, Yogita Rochlani

**Affiliations:** 1 Jacobi Medical Center and Albert Einstein College of Medicine, Bronx, New York, US; 2 Montefiore Medical Center and Albert Einstein College of Medicine, Bronx, New York, US

**Keywords:** cardiac sarcoidosis, cardiac FDG-PET, granulomatous myocarditis, inflammatory cardiomyopathy

## Abstract

Cardiac sarcoidosis (CS) is a widely underdiagnosed yet clinically significant form of granulomatous myocarditis associated with significant morbidity and mortality. Clinical presentation ranges from silent cardiac involvement detected on imaging to cardiomyopathy or sudden cardiac death. Diagnosis of CS remains challenging due to the lack of sensitivity and specificity of any single diagnostic method, underscoring the importance of elevated clinical suspicion and the use of multimodality imaging to guide diagnosis and treatment. In this review, we discuss the epidemiology, pathogenesis, clinical features, and diagnosis of this clinically evading and enigmatic disease.

## Introduction

Sarcoidosis is a multisystem granulomatous disease of unclear etiology that typically affects individuals between 25 and 60 years of age and is more common in women.^1^ The underlying cause is not entirely understood, but theories suggest that, in genetically susceptible individuals, an unknown antigenic trigger sets off an inflammatory cascade that results in granulomatous inflammation followed by fibrosis and scarring in some individuals.^1^ Environmental and genetic factors impact disease development and progression, resulting in geographic and racial differences in the prevalence and outcomes of affected patients.^1^ Although sarcoidosis can involve any organ system, it affects the lungs and thoracic lymph nodes in 90% of cases.^2^ Approximately 5% of patients have clinically manifest cardiac involvement, often presenting as conduction system abnormalities, ventricular arrhythmias, or cardiomyopathy, while asymptomatic cardiac involvement has been reported in up to 25% of cases.^2^

Diagnosis of cardiac sarcoidosis (CS) remains challenging as it requires a combination of clinical and radiological findings in addition to histologic evidence of noncaseating granulomas with exclusion of any other potential causes of such histologic findings.^3^ While its overall course depends on the organ systems involved, cardiac involvement is a prominent driver of morbidity and mortality. The prevalence of CS has been increasing over the past two decades, likely due to utilization of advanced cardiac imaging.^4^ However, CS continues to remain underdiagnosed as a reversible cause of cardiomyopathy and arrhythmias. In this review, we discuss the epidemiology, pathogenesis, clinical features, and diagnosis of CS with the purpose of increasing awareness about this disease since timely diagnosis and treatment can lead to better outcomes.

## Epidemiology

The incidence and prevalence of sarcoidosis varies widely across the globe and has been reported to be higher in populations of northern European and African American descents.^1^ The highest incidence has been reported in Scandinavian countries, with an estimated 11.5 cases per 100,000 individuals. In the United States (US), it ranges from 8 to 11 per 100,000 people and is reported to be lower in other parts of the world, including Canada (6.8 per 100,000) and East Asian countries such as South Korea (0.5-1.3 per 100,000).^5^ Similarly, reported prevalence ranges from 140 to 160 per 100,000 in Sweden and Canada, to 1 and 5 per 100,000 in South Korea, Taiwan and Japan.^5^ A US national healthcare database analysis between 2010 and 2013 revealed a three-times higher prevalence among Black Americans (141.4 per 100,000) compared with Whites (49.8 per 100,000), and a lower prevalence in Hispanics and Asians (21.7 vs 18.9 per 100,000, respectively). The highest prevalence was noted among Black American women (178.5 per 100,000).^6^ The Black Women’s Health Study estimated the average annual incidence among Black American women to be as high as 71 per 100,000 and a prevalence of nearly 2%.^7^ Mortality also is reported as higher among women and Black Americans.^8^

The lung is the most commonly involved organ system, occurring in more than 90% of patients with sarcoidosis,^9^ followed by extra-thoracic sites such as lymph nodes, skin, heart, spleen, liver, and other organs and tissues.^3^ Disease usually occurs in patients aged 25 to 45 years, with a second peak occurring in women > 50 years in Japan and European countries.^3,10^

Cardiac involvement can occur with multisystem sarcoidosis or may be the initial and only manifestation of the disease. Studies estimate 5% of patients with sarcoidosis have clinically manifest CS and up to a third have clinically silent disease as detected in imaging and autopsy studies.^2,3,4,11,12^ Moreover, up to half of the cases of cardiac involvement might present as isolated CS.^4^ Prevalence of CS has increased over the years as evidenced by studies in Finland showing a greater than 20-fold increase in detection between 1988 and 2012.^4^ Similarly, the US rate of transplantation for CS increased from 0.1% to 0.5% between 2011 and 2014.^13^ Use of advanced imaging modalities that aid prompt detection of CS potentially elucidates this increasing prevalence.

## Pathogenesis

Although the inciting antigenic trigger is yet to be identified, several factors are implicated in the pathogenesis of CS, including genetic susceptibility coupled with immune dysregulation, prior history of infections, and occupational or environmental factors. Human leukocyte antigen (HLA) alleles in the major histocompatibility complex gene are influential in the disease’s course. For example, HLA-DR17(3) is most associated with sarcoidosis in a White population, DRB1*03 is associated with spontaneous resolution, and HLA-DR15(2) or DR14(6) are associated with a chronic disease course.^14^ Implicated infectious agents include mycobacteria, Propionibacterium, Borrelia burgdorferi, Rickettsia helvetica, Epstein-Barr virus, and human herpes virus 8.^15^ Workers in lumbering and wood processing industries who are exposed to industrial organic dust showed elevated risk of sarcoidosis.^16^ Further clues on the role of environmental agents are drawn from observations indicating an increased incidence of sarcoidosis-like granulomatous pulmonary disease in World Trade Center survivors.^17,18^

Formation of discrete noncaseating granulomas consisting of epithelioid histiocytes and multinucleated giant cells surrounded by lymphocytes, plasma cells, and fibroblasts is the histological hallmark of sarcoidosis.^19^ The multinucleated giant cells are initially foreign body type (with haphazardly arranged nuclei) and later become Langhans type (with peripherally arranged nuclei). They may contain cytoplasmic inclusions, particularly Schaumann bodies or asteroid bodies.^20^ The immunological basis responsible for sarcoid generation is primarily driven by an exaggerated TH1 cell response with production of cytokines, including interleukin (IL)-2, interferonγ, and IL-12.^20,21^ This is followed by an anti-inflammatory TH2 type response marked by the presence of IL-4, IL-5, IL-10, and IL-13 cytokines.^20^ Interestingly, the development and disappearance of cardiac granulomas parallels the presence of these cytokines in the specified order.^22^ Fibrotic transformation of chronic granulomas is mediated by the switch to TH2 type response with contribution from platelet-derived growth factor-B, insulin-like growth factor 1, and insulin-like growth factor binding protein-related protein 2.^20,23^ Recent studies also demonstrate the role of Th17 cell mediated production of IFN-γ in pathogenesis of sarcoidosis and suggest imbalances in Tregs and Th17 cell response to be an added mechanism.^23,24^

In CS, myocardium is usually involved with endocardium or epicardium as an extension of the disease.^19,20,25^ Granulomas are frequently found in the basal interventricular septum, left ventricular (LV) free wall, right ventricle, and atria.^26,27,28,29^ With disease progression, the granulomatous inflammation elicits a repair response with scarring, different from the scarring seen in myocardial infarction, which is patchy rather than maximal in the subendocardial region or transmural.^20^ While distribution of granulomas in the interventricular septum results in conduction blocks that may be responsive to steroid therapy, patchy myocardial scarring provides a nidus for ventricular tachyarrhythmias, focal aneurysms, and systolic or diastolic LV dysfunction.

## Clinical Features of Cardiac Sarcoidosis

Cardiac sarcoidosis can present as (1) asymptomatic disease incidentally detected on screening in the setting of pulmonary/systemic sarcoidosis, (2) clinically manifest CS along with symptomatic or asymptomatic extra-CS, or (3) isolated CS. The cardinal manifestations of CS include high-grade atrioventricular (AV) blocks, ventricular tachycardias (VT) potentially resulting in sudden cardiac death (SCD), and cardiomyopathy. Rarer presentations include atrial arrhythmias, papillary muscle dysfunction and mitral regurgitation, constrictive pericarditis, pericardial effusion, or mimics of myocardial ischemia.^20,29,30^ Consequently, patients present with a history of dizziness, near-syncope or syncope, palpitations, fatigue, dyspnea, or other symptoms of congestive heart failure (HF).

Conduction abnormalities are the most common clinical manifestation of CS (12-62% of patients) and can present as bundle branch blocks (right or left), AV blocks of varying degrees, or even sinus arrest.^30^ These result from direct granulomatous inflammation of the septum involving the conduction system. Sustained or nonsustained VT has been reported in 2% to 42% patients with CS, while SCD may present in 12% to 65%.^30^ The most common mechanism of VT is re-entry from scar formation, while some studies also point to a role of active inflammation.^31,32^ Due to the patchy nature of the myocardial inflammatory process, multiple morphologies of VT also may be present. Occurrence of frequent premature ventricular contractions or polymorphic VT raises suspicion for active inflammation.^31^ VT often is the first manifestation of CS and predicts mortality.^32^

CS cardiomyopathy has a prevalence of 10% to 30% and can manifest as left, right, or biventricular-systolic or diastolic dysfunction in the early inflammatory or late fibrotic stages.^30,31^ Granulomatous infiltration of the myocardium can result in wall motion abnormalities in a noncoronary distribution, valvular regurgitation, LV dysfunction, LV wall thinning, aneurysmal dilation, scarring, or wall rupture. Moreover, reduced ventricular compliance from LV stiffening leads to diastolic dysfunction. Right ventricular (RV) dysfunction can result from granulomatous inflammation or more commonly as a consequence of LV dysfunction and sarcoid-induced pulmonary hypertension.^29^ One study showed a 3% incidence of HF in CS, twice that of the general population. Moreover, HF at presentation or RV involvement is associated with poor clinical outcomes.^4,33,34^

## Diagnosis of CS

Diagnosis of CS is clinically challenging due to the lack of sensitivity or specificity of any modality in isolation. Hence, multiple diagnostic tests need to be interpreted with the clinical context in mind. Tissue histology using endomyocardial biopsy remains the gold standard for definitive diagnosis. Advanced cardiac imaging techniques such as cardiac magnetic resonance (CMR) imaging and ^18^fluorine-fluorodeoxyglucose-postrion emission tomography (FDG-PET) have emerged as invaluable tools to aid disease detection as well as follow disease activity and response to treatment. Here we describe findings associated with CS on various testing modalities.

## Basic Cardiac Evaluation

### EKG Findings

Abnormalities on electrocardiography prompting suspicion and further imaging for CS include right bundle branch block, advanced or complete AV blocks, and ventricular arrhythmias, including ventricular tachycardia or fibrillation.^31^ Other nonspecific electrocardiogram (EKG) changes hinting at cardiac involvement in patients with sarcoidosis are axis deviation, abnormal Q waves (pseudoinfarct pattern), and ST changes in noncoronary patterns.^31,35^ VT can be the only presentation in sarcoidosis^36^ and can predict onset of heart failure in CS patients.^37^

### Echocardiography

Echocardiography plays an important role in screening for CS but has low sensitivity and specificity as a diagnostic tool.^38^ The most specific finding on echocardiography is basal ventricular septum thinning included as part of the Japanese Circulation Society (JCS) diagnostic criteria.^35^ It is defined as the thinning of the ventricular septum at 10 mm below the aortic annulus and/or the ratio of this measure to the thickness of the normal ventricular septum of ≤ 0.6.^35^ Other commonly reported abnormalities include ventricular aneurysm formation, myocardial thickening from edema or infiltration mimicking myocardial hypertrophy, unusual wall motion abnormalities in noncoronary patterns, and papillary muscle dysfunction.^38,39^ Moreover, echocardiography may also determine left atrial dysfunction, pulmonary hypertension, RV dysfunction, and the degree of diastolic dysfunction.^38,40^ Speckle tracking echocardiography can potentially enhance the yield of screening. A recent meta-analysis highlighted the utility of global longitudinal strain (GLS) imaging in predicting subclinical myocardial involvement and major cardiac events.^41^

### Biomarkers

Established markers of systemic sarcoidosis such as angiotensin converting enzyme (ACE) can be elevated in CS.^40^ Moreover, several known cardiac markers such as atrial natriuretic peptide, brain natriuretic peptide (BNP), high-sensitivity cardiac troponin T and I, and N-terminal proBNP have also been studied in CS.^40,42^ Interestingly, studies show that troponin levels can predict risk of arrhythmias, and levels often decrease with improvement in cardiac function and inflammation after treatment.^43,44,45^ Elevated BNP levels also have been reported as a marker of cardiac involvement in patients with extracardiac sarcoidosis.^45^ Markers of inflammation, including serum levels of soluble IL-2 receptors, neopterin and cytokines such as IL-1α, IL-2, IL-12 p40, and IFN-γ have been evaluated as markers of disease activity in small studies although sensitivity and specificity have been poor.^35,40^ The utility of biomarkers in clinical practice remains limited, and most are still being studied in research protocols.

## Advanced Cardiac Imaging for CS

### Cardiac Magnetic Resonance Imaging

CMR is considered the initial step in evaluation of CS due to its superior spatial resolution, ability to provide tissue characterization, and simultaneous structural and functional evaluation of the heart.^46,47^ In one study, CMR detected subclinical disease in 9.3% of asymptomatic patients without EKG abnormalities and in 4.7% of patients without any abnormalities at initial evaluation.^48^ Both acute and chronic stages can be discerned with different technical aspects of CMR. Late gadolinium enhancement (LGE) is the most reliable tool in CMR assessment. There is no pathognomonic distribution pattern for CS. However, studies show LGE in mainly subepicardial or midmyocardial areas of the basal septum and LV lateral wall with extension into the RV.^28,49,50,51^ Rarely, LGE also can be subendocardial, mimicking an infarct pattern.^49^ While LGE helps detect more chronic phases of diseases attributed to fibrosis or scarring, T2 mapping detects increased water content or edema in areas of active inflammation.^46^ A meta-analysis by Zhang et al. estimated the sensitivity and specificity for CS diagnosis by CMR at 95% and 92%, respectively.^52^ Among patients with extra-CS, CMR has shown diagnostic accuracy and independently predicted mortality, VT, and HF hospitalizations in those with cardiac symptoms and/or an abnormal EKG (HR 12.71; 95% CI, 1.48-109.35; *P* = .021).^48^ In patients with known or suspected CS, a pooled analysis of 760 patients showed that the presence of LGE in CMR increased likelihood of death from any cause and of future arrhythmogenic events.^53^

### FDG-PET

^18^FDG-PET imaging has become an important part of the multimodality approach used to diagnose and follow treatment response in CS. PET imaging serves as an indicator of ongoing inflammation. The principle follows uptake of ^18^FDG, a glucose analogue, into cells in macrophage-rich areas via the glucose transporter (GLUT); the GLUT is then metabolized to form ^18^FDG-6-phosphate, whose further intracellular metabolism via glycolytic pathway is terminated, resulting in metabolic trapping that enables detection using imaging techniques. The test involves suppressing glucose uptake in the normal myocardial cells, thus allowing for detection of uptake by inflammatory cells with increased metabolic activity. A joint expert consensus report of the Society of Nuclear Medicine and Molecular Imaging and American Society of Nuclear Cardiology recommend use of cardiac PET for CS diagnosis in the following clinical scenarios: (1) patients with histological evidence of extracardiac sarcoid or positive screening test for CS; (2) unexplained, new-onset significant conduction abnormality such as second- or third-degree AV conduction block in a patient < 60 years; (3) idiopathic sustained ventricular tachycardia; and (4) patients on treatment for CS to guide therapy.^54^ In the absence of recent whole-body PET imaging or the presence of clinical suspicion for extra-CS, a concomitant whole-body PET study should be performed, or at least imaging of chest, liver, and spleen to detect any extracardiac involvement that could be accessed to confirm a histologic diagnosis.^54^ Moreover, in patients in whom MRI is contraindicated and in advanced renal disease, PET can be a useful diagnostic tool.^55^

Inflammatory activity in CS using PET is interpreted both visually and quantitatively. Visual interpretation involves simultaneous evaluation for presence of myocardial perfusion defect (employing PET perfusion imaging or SPECT with either ^99m^Tc-labeled tracers or ^201^Tl) along with ^18^F-FDG uptake (***Table 1***). Uptake patterns on PET imaging suggestive of CS include focal and focal on diffuse uptake, whereas suppression of uptake or a diffuse pattern points to normal physiological uptake.^47,54,55^ Rest perfusion images are classified as normal or abnormal, with regional myocardial perfusion further categorized as mild, moderate, or severely reduced. Moreover, quantitative analysis using standard uptake value can add to the diagnostic utility, although thresholds for this are still under investigation.^56,57^ A recent systematic review showed that the sensitivity of ^18^F-FDG-PET in the diagnosis of CS ranges from 27% to 100% with a similar specificity (28-100%) and is dependent on disease activity.^47^ A meta-analysis by Youssef et al. evaluating ^18^FDG-PET imaging for CS revealed a sensitivity and specificity of 89% and 78%, respectively.^58^ The presence of both perfusion and metabolic abnormality on PET and focal RV uptake has been associated with a 3-fold increase in adverse events including death or VT.^33^ In addition to RV involvement, uptake in basal anterolateral area of LV in steroid-naïve patients was predictive of adverse outcomes.^59^

**Table 1:** Features of cardiac sarcoidosis as shown on ^18^fluorine-fluorodeoxyglucose-postrion emission tomography. Adapted from Blankstein et al. J Am Coll Cardiol. 2014.^33^ FDG: ^18^fluorine-fluorodeoxyglucose


FDG UPTAKE	REST PERFUSION	INTERPRETATION

**Normal metabolism and perfusion**		

None	Normal	Normal study

Diffuse uptake (nonspecific)	Normal	Inadequate myocardial glucose suppression

**Abnormal metabolism or perfusion**		

Focal uptake	Normal	Early disease or normal variant

None	Perfusion defect	Scar from any etiology

**Abnormal metabolism and perfusion**		

Focal uptake	Perfusion defect in area of focal FDG uptake	Inflammation + scar in the same area

Focal uptake	Perfusion defect in area separate from FDG uptake	Inflammation + scar in different areas

Focal on diffuse uptake	Multiple perfusion defects	Diffuse inflammation or inflammation + inadequate glucose suppression and scar


The diagnostic accuracy of PET imaging can be limited by lack of suppression of uptake in normal tissue or false positives due to other inflammatory conditions, ischemia, or hibernating myocardium, leading to confounding results. This can be avoided by adhering to preparation protocols, including a low-carbohydrate, fat-rich diet (such as Atkin’s diet) the day before the study followed by at least 12 to 18 hours of fasting before testing.^33,57^

### CMR Versus FDG-PET Imaging for CS

Advanced imaging modalities help increase the diagnostic yield, bear prognostic implications, and help direct therapy. CMR and ^18^FDG-PET imaging have a complementary role in the diagnosis and management of CS since both evaluate slightly different aspects of the disease. The greater specificity and negative predictive value of CMR combined with its ability to help rule out other diagnoses make it an ideal initial screening test.^46^ Imaging with FDG-PET/CT is warranted in equivocal or negative CMR findings in the setting of high clinical suspicion or in cases with CMR findings with highly probable CS to detect active inflammation and plan further therapy.^47,49^ Visual reduction in the intensity and extent of myocardial inflammation on follow-up ^18^FDG-PET imaging and indices, such as Standard Uptake Value, show utility in monitoring response to therapy.^60,61,62^ The exact timing to repeat PET imaging is unknown; however, 4 to 6 months post initiation of treatment has been suggested.^38^ Moreover, the Heart Rhythm Society (HRS) indicates use of CMR to determine eligibility for implantable cardioverter defibrillator (ICD) placement with indications being reduced LV ejection fraction (35%) after immunosuppressive therapy (Class I) or if LGE is present in patients with LV ejection fraction 35% to 49% after immunosuppression (Class IIb).^3^

### Endomyocardial Biopsy

The identification of epithelioid noncaseating granulomas in cardiac tissue specimen is considered definitive for diagnosis of CS after exclusion of alternative diagnoses. However, the diagnostic yield of electromyocardial biopsy (EMB) in CS is low, estimated at 20% to 30%.^63,64,65^ Several factors impact yield, including technique, the patchy distribution of lesions having increased predilection for left heart favoring midmyocardial and subepicardial areas, and stage of disease.^66^ If performed during chronic stages, granulomas and lymphocytic infiltration tend to disappear and are replaced by fibrosis.^35^ EMB is required for confirmation of diagnosis in absence of extracardiac histological diagnosis and isolated cardiac sarcoidosis and to rule out other causes of fulminant cardiomyopathy, such as giant cell myocarditis. In addition to histological evaluation, concurrent staining with Grocott staining for fungal infection and Ziehl-Neelsen staining for acid-fast microorganisms should be conducted as indicated.^35^ The presence of epithelioid giant cells in myocardial biopsy samples is strongly suggestive of cardiac sarcoidosis.^35^ Electroanatomic mapping-guided EMB helps increase diagnostic yield up to 41%, with additional benefit from cardiac imaging.^64,66^ The prognostic yield of EMB remains uncertain, with different studies providing contrasting results.^65,66^

### Testing for Extracardiac Sarcoidosis

Evaluation for extracardiac involvement in patients with suspected CS is crucial and may often be the only feasible approach to establishing a histologic diagnosis. Chest radiography or high-resolution computed tomography (HRCT) can demonstrate bilateral hilar or mediastinal lymphadenopathy along with lung parenchymal findings of nodular and patchy ground-glass opacities, pale infiltrates, and cyst formation that distribute along the lymphatic vessels, suggestive of pulmonary sarcoidosis. HRCT in pulmonary sarcoidosis also demonstrates very fine to fine nodular shadows along the peribronchovascular sheaths, interlobular septa and pleural surface, and tracheal wall thickening, while more advanced cases develop fibrosis and traction bronchiectasis.^67^ Lung or lymph node biopsies obtained through transbronchial needle aspiration (TBNA), endobronchial biopsy, transbronchial lung biopsy, and, more recently, endosonography with nodal aspiration, either endobronchial ultrasound-guided TBNA or esophageal ultrasonography with fine-needle aspiration, can aid in pathological confirmation of diagnosis. Depending on the system involved, cutaneous biopsies, eye examination, and further imaging may be indicated.^35,67^

## Diagnostic Criteria for CS

**Figure 1:**
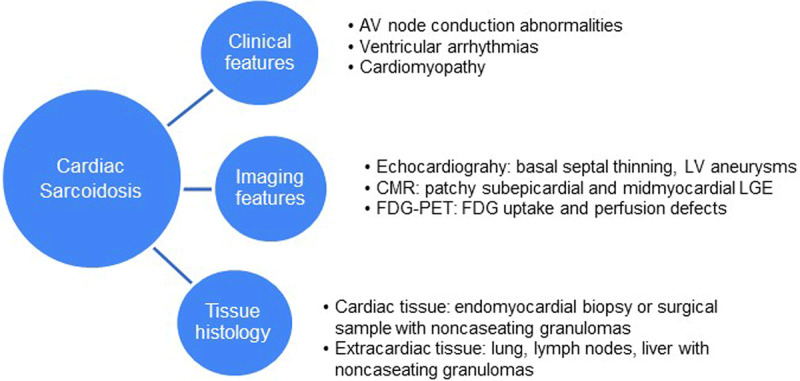
Diagnosis of cardiac sarcoidosis. LV: left ventricular; CMR: cardiac magnetic resonance; LGE: late gadolinium enhancement; FDG-PET: fluorine-fluorodeoxyglucose-positron emission tomography

Diagnosis of sarcoidosis requires a compatible clinical and radiologic presentation, pathologic evidence of noncaseating granulomas, and exclusion of other diseases with similar findings (***Figure 1***).^68^ Since histologic evidence of noncaseating granulomas in the myocardium can be difficult to establish, CS is typically diagnosed based on clinicopathological findings in conjunction with imaging and extracardiac tissue histology. Three sets of diagnostic criteria have been prescribed by professional societies based on expert consensus. Diagnostic criteria commonly used in practice include the HRS criteria and the Japanese Ministry of Health and Welfare/JCS criteria (***Table 2***).

**Table 2:** Summary of Heart Rhythm Society and Japanese Circulation Society guidelines for diagnosis of CS. CS: cardiac sarcoidosis; LVEF: left ventricular ejection fraction: VT: ventricular tachycardia; VF: ventricular fibrillation; PET: positron emission tomography; CMR: cardiac magnetic resonance; ^18^F-FDG-PET: ^18^fluorine-fluorodeoxyglucose-postrion emission tomography; EKG: echocardiographic Adapted from^3,35,42^


HEART RHYTHM SOCIETY GUIDELINES	JAPANESE CIRCULATION SOCIETY GUIDELINES

**Definite or histological diagnosis:** Requires presence of noncaseating granulomas myocardial tissue and absence of alternative cause A. Probable* or clinical diagnosis: Histological diagnosis of extracardiac sarcoidosis **AND** Presence of ≥ 1 of the following: Steroid ± immunosuppressant responsive cardiomyopathy or heart block Unexplained reduced LVEF (< 40%) Unexplained sustained (spontaneous or induced) VT Mobitz type II 2nd degree heart block or 3rd degree heart block Patchy uptake on dedicated cardiac PET (in a pattern consistent with CS) Late gadolinium enhancement on CMR (in a pattern consistent with CS) Positive gallium uptake (in a pattern consistent with CS) **AND** B. Other causes for the cardiac manifestation(s) have been reasonably excluded *Probable is considered adequate to establish a clinical diagnosis of CS	**1) Histological diagnosis group:** Requires biopsy findings demonstrating noncaseating epithelioid granulomas from endomyocardial biopsy or surgical cardiac specimens **2) Clinical diagnosis group: (negative myocardial biopsy or not undergoing myocardial biopsy)** **A)** Extracardiac biopsy proven sarcoid AND clinical findings strongly suggestive of cardiac involvement** **OR** **B)** Clinical findings strongly suggestive of pulmonary or ophthalmic sarcoid **AND** At least 2 out of 5 characteristic lab findings of sarcoidosis*** AND Clinical findings strongly suggestive of cardiac involvement**

	**Clinical findings defining cardiac involvement 1) Two or more of the five major criteria (a) to (e) are satisfied 2) One of the five major criteria (a) to (e) and two or more of the three minor criteria (f) to (h) are satisfied

	Criteria for cardiac involvement of sarcoidosis **1. Major criteria** (a) High-grade atrioventricular block or VT/VF (b) Basal thinning of the ventricular septum or abnormal ventricular wall anatomy (ventricular aneurysm, thinning of the middle or upper ventricular septum, regional ventricular wall thickening) (c) Left ventricular contractile dysfunction (LVEF < 50%) (d) ^67^Ga citrate scintigraphy or ^18^F-^18^FDG-PET reveals abnormally high tracer accumulation in the heart (e) Gadolinium-enhanced MRI reveals delayed contrast enhancement of the myocardium **2. Minor criteria** (f) Abnormal EKG findings: ventricular arrhythmias (nonsustained ventricular tachycardia, multifocal or frequent premature ventricular contractions), bundle branch block, axis deviation, or abnormal Q waves (g) Perfusion defects on myocardial perfusion scintigraphy (h) Endomyocardial biopsy: monocyte infiltration and moderate or severe myocardial interstitial fibrosis


*** Clinical diagnosis of sarcoidosis is supported when at least two of the five characteristic findings are observed. Bilateral hilar lymphadenopathy High serum angiotensin-converting enzyme activity or elevated serum lysozyme levels High serum soluble interleukin-2 receptor levels Significant tracer accumulation in 67Ga citrate scintigraphy or ^18^FDG-PET A high percentage of lymphocytes with a CD4/CD8 ratio of > 3.5 in BAL fluid

The HRS recommendations described criteria for “definite” or histological, requiring myocardial biopsy findings, and “probable” or clinical diagnosis, necessitating biopsy proven sarcoid in an extracardiac site.^3^ In 2014, the World Association of Sarcoidosis and Other Granulomatous Disorders (WASOG) revised the 1999 WASOG Sarcoidosis Organ Assessment Instrument, which replaced the dated ACCESS (A Case Control Etiology of Sarcoidosis Study) sarcoidosis organ assessment instrument. Based on expert opinion consensus, they categorized the likelihood of organ involvement as either (A) highly probable; likelihood of sarcoidosis causing this manifestation of at least 90%; (B) probable; likelihood of sarcoidosis causing this manifestation of between 50% and 90%; or (C) possible; likelihood of sarcoidosis causing this manifestation of less than 50%.^69^ In 2017, the JCS further revised their 2006 criteria to guide diagnosis based on a histological or clinical basis.^35,42^ Important diagnostic clues such as fatal ventricular arrhythmia (sustained ventricular tachycardia and ventricular fibrillation), abnormal ventricular anatomy, and advanced cardiac imaging findings (CMR, FDG-PET) were incorporated into major criteria, reflecting the significance of these manifestations in CS.^42^ Moreover, diagnosis in the clinical group did not mandate biopsy findings of sarcoidosis from cardiac or extracardiac tissue, a significant change from previous recommendations. The 2017 JCS guidelines also prescribe criteria to diagnose isolated CS.^35,42^

## Isolated Cardiac Sarcoidosis

Isolated CS (iCS) is characterized by cardiac involvement in the absence of clinical, histological, or imaging findings of extracardiac disease.^70^ Recognizing iCS as a distinct clinical entity, the JCS laid out criteria for clinical or histological diagnosis (***Table 3***).^35^ Reported prevalence ranges widely from 3.2% to 54%. ***Table 4*** summarizes characteristics of studies on iCS published after 2010.^71,72,73,74,75,76,77^ Patients with iCS usually present with advanced heart disease and have worse survival outcomes, explained by late diagnosis.^4^ iCS patients present with LV systolic dysfunction or sudden cardiac death more often than patients with extracardiac sarcoidosis with cardiac involvement.^64^ While EMB confers definitive diagnosis, advanced imaging modalities are preferred for their higher yield (95% and 74% sensitivity for CMR and FDG-PET, respectively), and aiding extracardiac sarcoidosis detection. Signs of extracardiac sarcoidosis such as lymphadenopathy can manifest later, although its relevance to treatment and prognosis is unclear.^70^

**Table 3:** Japanese Circulation Society (JCS) guidelines for isolated cardiac sarcoidosis.^35,42^


PREREQUISITE CRITERIA	

1. No clinical findings characteristic of sarcoidosis are observed in any organs other than the heart. (The patient should be examined in detail for respiratory, ophthalmic, and skin involvements of sarcoidosis. When the patient is symptomatic, other etiologies that can affect the corresponding organs must be ruled out.) 2. 67Ga scintigraphy or ^18^FDG-PET reveals no abnormal tracer accumulation in any organs other than the heart. 3. A chest CT scan reveals no shadow along the lymphatic tracts in the lungs or no hilar and mediastinal lymphadenopathy (minor axis > 10 mm).	

**CLINICAL DIAGNOSIS GROUP**	**HISTOLOGICAL DIAGNOSIS GROUP**

Prerequisite criteria **AND** ≥ three other criteria of the major criteria (a)-(e) are satisfied per JCS guidelines, Table 2*	Demonstration of noncaseating epithelioid granulomas in endomyocardial biopsy or surgical specimens


^18^FDG-PET: ^18^fluorine fluorodeoxyglucose-positron emission tomography; CT: computed tomography * Refer JCS guidelines in ***Table 2*** for major criteria a-e

**Table 4:** Prevalence of isolated cardiac sarcoidosis.^71,72,73,74,75,76,77^ AV block: atrioventricular block; CMR: cardiac magnetic resonance; PET: positron emission tomography; EMB: endomyocardial biopsy; HF: heart failure; HRS: Heart Rhythm Society; JHMW: JHM strain of mouse hepatitis virus; LN: lymph node; VA: ventricular arrhythmias; VF: ventricular fibrillation; VT: ventricular tachycardia; ^18^FDG-PET: ^18^fluorine-fluorodeoxyglucose PET; WASOG: World Association for Sarcoidosis and Other Granulomatous Diseases


AUTHOR	STUDY PERIOD	N	STUDY POPULATION	COUNTRY	STUDY POPULATION CLINICAL MANIFESTATIONS N (%)	DIAGNOSTIC MODALITIES (N)	PREVALENCE n (%)	MEDIAN AGE	SEX MALE

Kandolin et al.	1998–2014	110	Patients diagnosed with CS	Finland	AV block 48 (44) VT/VF 36 (33) HF 20 (18)	EMB (55/92) Explant (6) Autopsy (2) mediastinal LN biopsy (18) Whole body PET 31 CMR 59 PET 66 CMR and/or PET (38) Other (9)	Definite: 59/110 (54)	51 ± 9	39

Tezuka et al.	1995–2008	83	Patients with clinical sarcoidosis	Japan	*N = 15* VA 8 (53)VT 7 (47)VF 1 (7)Automatic ICD 9 (60)Complete AV block 3 (20) HF 6 (40)	JCS Criteria CMR FDG-PET EMB	Definite: 11/41 (27)	63.5 ± 15.9*	7*

Simonen et al.	2005–2013	68	Patients with known CS	Finland	Complete AV block 37 (54) Sustained VT 18 (26) HF 7 (10) VF 4 (6) PVCs 2 (2.3)	Cardiac ^18^FDG PET (68) CMR (43) EMB (56) Whole body ^18^FDG PET (n = 57) Mediastinal LN biopsy (24)	Definite: 13/57 (23)	50 ± 9	21

Juneau et al.	2017	31	Patients first presenting with clinically manifest CS	Canada	High-degree AV block 18 (58) VT or cardiac arrest 6 (19) High-degree AB block/VT 3 (10) HF 3 (10) Other 1 (3)	Cardiac and whole body ^18^FDG-PET-CT	Definite: 1/31 (3.2)	56 ± 8	14

Giudicatti et al.	2007–2018	52	All cases of proven or probable CS based on HRS and local consensus	Australia	HF 17 (53) VF/VT 7 (22) ICD 21 (66)	Cardiac and whole body ^18^FDG-PET-CT	Definite: 3/32 (9.4)	59	22

Kawai H	2013–2019	94	All patients with suspected CS	Japan	N = 7* Sustained VT/VF or high-degree conduction block 4 (57) HF 7 (100)	Cardiac and whole body ^18^FDG-PET-CT	Clinical: 7/34 (21) Based on JCS guidelines	59.4 ± 14.9*	4*

Sperry BW	2002–2014	27	EMB-proven CS	USA	High-degree AV block 15 (56) HF 27 (100) VT 16 (59)	^18^FDG-PET-CT (16) CMR (12)	Definite: 14/27 (52)	53.8 ± 9.5	16

Chazal et al.	2000–2017	15	CS in explanted hearts/ATS- or WASOG-based diagnosis of sarcoidosis	France	VT 7 (47) VF 1 (7) Complete AV block 3 (20) HF 6 (40)	Echocardiography CMR (6) FDG-PET (2)	Definite: 3/15 (20)	48	10


* In iCS group.

## Clinical Entities Confounding Cardiac Sarcoidosis Diagnosis

Several conditions mimic CS presentation. These differential diagnoses can be clarified using histology or imaging modalities.^20,78,79,80^ Differentiation is vital because it carries implications in treatment and prognosis. ***Table 5*** lists the differential diagnosis one must keep in mind when ruling in or out sarcoidosis.

**Table 5:** Clinical, imaging, and histological features of diagnostic confounders.^20,78,79,80^


DISEASE	CLINICAL FEATURES	HISTOLOGY	IMAGING

Giant cell myocarditis	Ventricular arrhythmias Complete heart block Rapidly progressive heart failure, shock	Lack of granuloma formation Inflammatory infiltrate of eosinophils, lymphocytes, macrophages, and giant cells associated with myocyte necrosis	Echocardiographic findings: wall thickening, normal or enlarged LV size, decreased LV systolic function with acute progression to LV dilation and decreased LVEF

Idiopathic dilated cardiomyopathy	Heart failure, arrhythmia Positive family history	Myocyte hypertrophy and replacement fibrosis with variable involvement of the conduction system	Dilated LV with global ventricular dysfunction Linear stripe of LGE in ventricular septum on MRI or no LGE

Arrhythmogenic RV cardiomyopathy	Ventricular arrhythmias RV failure Family history Sudden cardiac death	Transmural fibrofatty replacement of myocardium	RV dilatation and dysfunction Fibrofatty infiltration of RV Dyskinesia of RV free wall with RV aneurysms

Amyloidosis	Heart failure Heart block Atrial fibrillation Multiple myeloma, renal insufficiency and/or nephrotic syndrome	Amorphous hyaline deposits seen predominantly in the extracellular space Typical apple-green birefringence with Congo red dye under polarized light microscopy and unique cross–β-pleated sheets under electron microscopy	Biventricular hypertrophy including valves and RV Biatrial enlargement Diffuse nulling abnormality of myocardium on MRI with LGE

Hypertrophic cardiomyopathy	Heart failure Ventricular arrhythmias Atrial fibrillation Family history	Myocyte hypertrophy and disarray Presence of interstitial and replacement fibrosis	LV hypertrophy > 15 mm (often asymmetric) Scattered patchy midmyocardial scar on MRI and LGE predominant in RV insertion points of ventricular septum

Myocarditis (tuberculous, fungal, bacterial, viral)	Heart failure Chest pain Atrial and ventricular arrhythmias Clinical features of causative organism	Necrotizing granulomas in case of TB, disseminated fungal infection Demonstration of implicated organisms on staining Myofiber necrosis Neutrophilic or mononuclear infiltrate Chronic stages: fibrosis with disruption of the normal myocardial architecture	Patchy epicardial LGE Edema on MRI


LV: left ventricular; LVEF: LV ejection fraction; LGE: late gadolinium enhancement; MRI: magnetic resonance imaging; RV: right ventricular

## Practical Approach to Diagnosing CS

**Figure 2:**
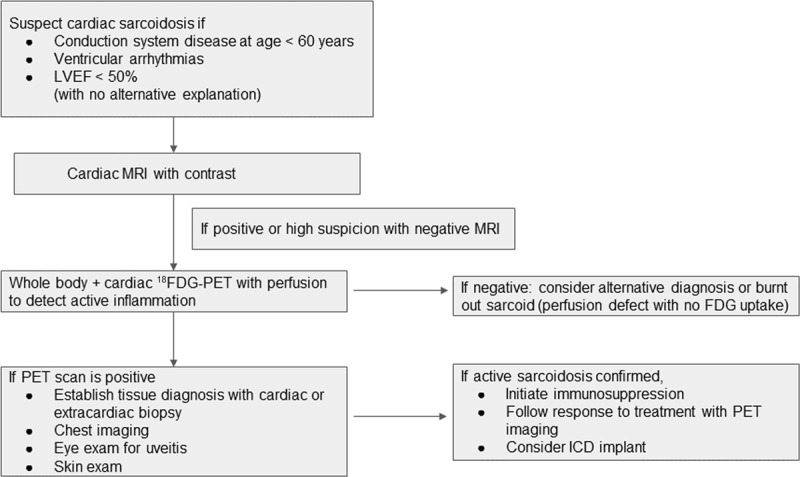
Approach to diagnosis for suspected cardiac sarcoidosis. LVEF: left ventricular ejection fraction; MRI: magnetic resonance imaging; ^18^FDG-PET: ^18^fluorine-fluorodeoxyglucose postrion emission tomography; PET: positron emission tomography; ICD: implantable cardioverter defibrillator

**Figure 3:**
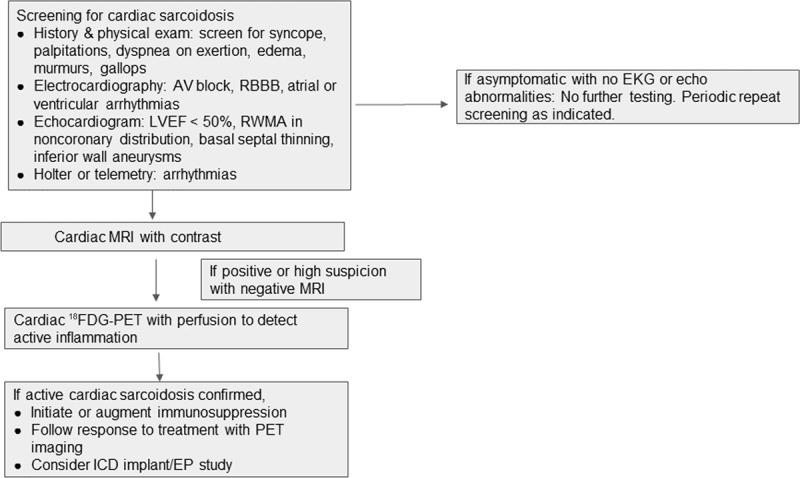
Approach to screening for patients with extracardiac sarcoidosis. AV: atrioventricular; RBBB: right bundle branch block; LVEF: left ventricular ejection fraction; RWMA: regional wall motion abnormalities; MRI: magnetic resonance imaging; ^18^FDG-PET: ^18^fluorine-fluorodeoxyglucose postrion emission tomography; PET: positron emission tomography; ICD: implantable cardioverter defibrillator; EP: electrophysiological; EKG: electrocardiogram: echo: echocardiogram

CS remains a challenge to diagnose, and diagnostic testing needs to be tailored to the clinical scenario in which it is being used. Clinicians may encounter three main clinical scenarios that trigger further testing for CS: (1) patients with clinical features suggestive of CS, ie, new heart block under age 60 years, nonischemic cardiomyopathy with electrocardiographic or echocardiographic features suggestive of CS, or idiopathic VT with known extracardiac sarcoidosis; (2) patients with clinical features suggestive of CS without known extracardiac sarcoidosis; and (3) patients with known extracardiac sarcoidosis without clinical features of cardiac involvement. We suggest a practical approach to diagnostic testing in these scenarios as shown in ***Figures 2*** and ***3***.

In patients with suspected CS based on their clinical presentation, CMR remains the initial test of choice and can be used to guide further testing with ^18^FDG-PET. The HRS guidelines recommend screening for CS in patients with a history of extracardiac sarcoidosis and those under 60 years presenting with unexplained Mobitz II or third-degree AV block and VT of unknown etiology.^3^ In patients with previously diagnosed extracardiac sarcoidosis, the diagnosis of CS can be made with relative ease if clinical features and imaging findings consistent with CS are present. The challenge often arises in patients with a clinical syndrome suspicious for CS but without a diagnosis of extracardiac sarcoidosis. Prior to labeling these cases as isolated cardiac sarcoidosis, a thorough search for extracardiac sarcoidosis must be undertaken with whole body PET imaging, skin exam, eye exam for uveitis, and biopsies if lymph node, lung, or liver involvement are suspected. In cases of isolated cardiac sarcoidosis, in addition to searching for extracardiac involvement, a comprehensive search for any other potential clinical mimics or causes of myocarditis must be performed.

It is suggested that patients with known extracardiac sarcoidosis without symptomatic cardiac involvement undergo periodic assessment for CS with appropriate history and EKG.^48^ A recent large observational study assessing cardiac outcomes in 12,042 sarcoidosis patients (without prior CS diagnosis) noted a higher risk of cardiovascular events, including incident HF, HF-related death, and composite outcomes inclusive of ICD implantation, ventricular arrhythmias, and cardiac arrest. This affirms the necessity of early recognition of CS in this population.^81^ Utilization of advanced imaging techniques in this population remains debated outside of research studies due to limited knowledge regarding the impact of treatment in asymptomatic individuals, particularly given the risks of immunosuppression.

## Conclusion

CS is an under-recognized cause of nonischemic cardiomyopathy and arrhythmias. CS can present as the initial, or even the only, manifestation of sarcoidosis and is associated with adverse outcomes. Diagnosis of CS remains challenging and requires a combination of clinical, radiologic, and histologic evidence. Advanced imaging techniques including CMR and FDG-PET are increasingly being used for diagnosis and guiding treatment in CS. Additional studies are required to improve diagnostic accuracy in cases of isolated cardiac sarcoidosis and to inform screening guidelines and the care of patients with asymptomatic cardiac involvement.

## Key Points

- Cardiac sarcoidosis is an under-recognized but treatable cause of cardiomyopathy and arrhythmias.
- Diagnosis of cardiac sarcoidosis is challenging and requires a combination of clinical, radiologic, and histologic findings.
- Endomyocardial biopsies have low sensitivity for diagnosis of cardiac sarcoidosis, and use of imaging or electroanatomic mapping guidance can help improve diagnostic yield.
- Clinical vigilance for cardiac sarcoidosis is important due to high risk of morbidity and mortality if left untreated.
